# Clonal Cytogenetic Evolution in Relapse of Myeloid Hematological Neoplasms After Allogeneic Stem Cell Transplantation

**DOI:** 10.3390/cancers18101665

**Published:** 2026-05-21

**Authors:** Emin Abdullayev, Julia Pross, Lejla Caluk Klacar, Shirneshan Katayoon, Laurentiu-Doru Filip, Anna Ossami Saidy, Thomas Held, Bertram Glaß, Snjezana Janjetovic

**Affiliations:** 1 Department of Hematology and Cell Therapy, HELIOS Klinikum Berlin-Buch, 13125 Berlin, Germany; emin.abdullayev@helios-gesundheit.de (E.A.); julia.pross@helios-gesundheit.de (J.P.); lejla.calukklacar@helios-gesundheit.de (L.C.K.); laurentiu-doru.filip@helios-gesundheit.de (L.-D.F.); anna.ossami-saidy@helios-gesundheit.de (A.O.S.); thomas.held@helios-gesundheit.de (T.H.); bertram.glass@helios-gesundheit.de (B.G.); 2 Department of Hematology and Medical Oncology, INDIGHO Laboratory, University Medical Center Göttingen (UMG), 37075 Göttingen, Germany; shirneshan@med.uni-goettingen.de

**Keywords:** hematologic malignancies, allogeneic hematopoietic stem cell transplantation (allo-HSCT), chromosome banding analysis, cytogenetic abnormality, clonal evolution

## Abstract

**Background:** Relapse is the leading cause of treatment failure in patients with myeloid hematologic malignancies undergoing allogeneic hematopoietic cell transplantation. Clonal genomic evolution may contribute to post-transplant relapse, yet its determinants and prognostic impact remain incompletely characterized. **Methods:** In this retrospective study, we analyzed 63 patients with myeloid neoplasms who underwent cytogenetic evaluation both at diagnosis and at relapse after allogeneic hematopoietic stem cell transplantation. Cytogenetic changes (CGE), including evolution, devolution, or combined patterns, were assessed and correlated with clinical characteristics, prior treatment exposure, and survival outcomes. **Results:** Cytogenetic changes were observed in 46.1% of patients. The presence of cytogenetic changes (CGE) was strongly associated with the presence and complexity of cytogenetic abnormalities at initial diagnosis, whereas prior chemotherapy exposure, conditioning intensity, and donor type showed no significant association. Patients with cytogenetic changes had a lower complete remission rate at day 30 after transplantation; however, relapse-free survival and post-relapse survival did not differ significantly between groups. **Conclusions:** These findings suggest a potential association between post-transplant cytogenetic changes and intrinsic genomic instability, although treatment-related effects cannot be excluded. Larger, disease-stratified studies integrating cytogenetic and molecular analyses are warranted to further clarify the biological and prognostic relevance of clonal evolution following transplantation.

## Simple Summary

Disease recurrence after allogeneic stem cell transplantation remains the principal cause of treatment failure in patients with myeloid malignancies. One possible explanation is the acquisition of additional genetic alterations by malignant cells over time. In this study, we investigated the emergence of cytogenetic changes at relapse and their association with prior treatment exposure and clinical outcomes. Nearly half of the patients developed cytogenetic changes at relapse. These alterations occurred more frequently in individuals with complex cytogenetic abnormalities at diagnosis, suggesting that an unstable genomic background may predispose further clonal diversification. In contrast, prior chemotherapy, conditioning regimen, and donor type were not associated with the emergence of new abnormalities. Although patients with cytogenetic changes showed a lower early response rate, long-term survival outcomes were not significantly affected. Overall, our findings suggest that cytogenetic alterations at relapse may primarily be driven by disease-intrinsic biological features rather than treatment-related genomic damage.

## 1. Introduction

Cytogenetics plays an important role in the diagnosis, classification, and prognosis of various hematologic malignancies [1,2,3,4,5,6]. However, cytogenetic abnormalities detected at the initial diagnosis may change during disease progression or relapse [7,8,9,10,11,12,13,14,15,16,17,18], indicating the development of a new subclone [7,8,9,10,11,12,13,14,15,16,17,18].

Karyotype evolution has been observed in patients with acute myeloid leukemia (AML) at relapse after chemotherapy, where it has been associated with shorter remission duration and inferior clinical outcomes [7,8,9,10,13,14,17,19,20]. More recently, studies in relapsed and refractory AML have underscored that clonal evolution represents a biological driver of disease progression and treatment resistance rather than merely a descriptive cytogenetic phenomenon [13]. Similarly, in myelodysplastic syndromes (MDS), clonal cytogenetic evolution has been linked to inferior overall survival and increased leukemic transformation rates highlighting the dynamic biological impact of cytogenetic progression [14,21,22].

Evidence from other aggressive hematologic malignancies such as Burkitt lymphoma further suggests that disease progression and relapse are accompanied by a marked increase in cytogenetic complexity and predominantly linear clonal evolution, supporting the concept that secondary chromosomal aberrations may contribute to treatment resistance and adverse outcomes [17].

Patients undergoing allogeneic hematopoietic stem cell transplantation (allo-HSCT) have a higher frequency of clonal karyotype evolution at relapse compared with those treated with conventional chemotherapy [11].

However, very limited data exist regarding a potential association between the dose or specific types of chemotherapy at the initial diagnosis and the development of cytogenetic changes at relapse after allo-HSCT [9,11,16]. Therefore, we analyzed sequential karyotypes at relapse after allo-HSCT in patients with myeloid disease treated at our center to further characterize the patterns of cytogenetic changes and their clinical impact.

In addition, we aimed to explore the potential influence of prior therapy and conditioning regimens on clonal cytogenetic changes at relapse. Finally, we aimed to investigate the correlation between the acquisition of new cytogenetic changes at relapse and overall survival.

## 2. Materials and Methods

### 2.1. Study Design and Patient Cohort

We conducted a retrospective, single-center cohort study at the Clinic for Hematology and Cell Therapy, Helios Clinic Berlin Buch, Germany. The study included all patients with AML, MDS, or myeloproliferative neoplasms (MPN) who relapsed after receiving an allo-HSCT between January 2015 and August 2024. Only patients with available karyotype analysis both at the initial diagnosis and at relapse after allo-HSCT were included in to the study. Cytogenetic comparisons were based on the karyotype at initial diagnosis and at relapse after allo-HSCT, as this reflects the baseline cytogenetic profile prior to subsequent therapies and transplantation.

This patient selection was applied to enable a focused analysis of cytogenetic changes at relapse based on paired karyotypic data, in line with the study objective to investigate clonal evolution rather than predictors of relapse.

Of the 511 patients analyzed, 63 met the inclusion criteria. Diagnosis and risk stratification of hematologic neoplasms were performed according to the 2017 and 2022 WHO classification [2,23]. Additionally, the risk score was calculated according to the ELN 2017 and 2022, DIPSS, and IPSS-R classification systems (Tables S1 and S2) [1,24,25,26]. Karyotypic changes were categorized as follows: cytogenetic evolution, defined as the emergence of one or more clonal cytogenetic abnormalities in addition to a pre-existing karyotype; cytogenetic devolution, defined as the loss of one or more clonal cytogenetic abnormalities present in the initial karyotype; and combined evolution/devolution, defined as the simultaneous loss of pre-existing clonal abnormalities and acquisition of new clonal cytogenetic abnormalities [16].

The patients were stratified into two groups.

The first group (cytogenetic clonal evolution (CGE) group) included patients who exhibited any form of cytogenetic change at relapse, including cytogenetic evolution, devolution, or combined evolution/devolution, while the second group (non-CGE group) comprised patients with no detectable changes compared with the initial karyotype.

Both groups were compared with respect to age at initial diagnosis of hematological neoplasia, age at time of allogeneic transplantation, therapy prior to allogeneic transplantation, type of conditioning and donor, response after allogeneic transplantation at day 30, mean time (in months) to relapse and number of cytogenetic changes at initial diagnosis and in recurrence of the disease after allo-HSCT.

Molecular profiling by next-generation sequencing was deliberately not included, as only a limited subset of mutations is currently standardized for MRD assessment [27], and broader NGS data would have introduced heterogeneity and limited interpretability; therefore, the analysis was restricted to cytogenetics.

The local ethics committee of the city of Berlin, Germany (Eth17-23) approved this study. Due to the retrospective design, the requirement for informed patient consent was waived.

### 2.2. Data Collection

Demographic and clinical patient characteristics were retrieved from electronic medical records. Collected variables included sex, age at diagnosis of the hematologic neoplasia, type of hematologic neoplasia, age at allo-HSCT, time to relapse after allo-HSCT, post-relapse survival, type of therapy administered before allo-HSCT, conditioning regimen, donor type, response at day 30 after allo-HSCT, number of cytogenetic abnormalities at initial diagnosis, and overall clinical outcome.

### 2.3. Standard Cytogenetic Analyses

Karyotyping was performed using the standard G-banding technique. Briefly, bone marrow cells were cultured for 24 or 48 h in RPMI 1640 medium (Gibco BRL, New York, NY, USA) supplemented with 5% fetal bovine serum (Gibco BRL, New York, NY, USA) at 37 °C, using mitotic stimulants containing granulocyte colony-stimulating factor (G-CSF), interleukin-1a, interleukin-3, and erythropoietin (EPO). Chromosomes were visualized using the trypsin–Giemsa method and analyzed with Ikaros Karyotyping Software (MetaSystems, Altlussheim, Baden-Württemberg, Germany). Karyotypes were classified according to the International System for Human Cytogenomic Nomenclature (ISCN 2016 and 2020) [28,29]. Donor- and recipient-derived metaphases were distinguished based on sex chromosome differences in sex-mismatched transplants. In sex-matched cases, relapse-associated metaphases were identified by the presence of previously documented clonal cytogenetic abnormalities.

### 2.4. Fluorescence in Situ Hybridization

Fluorescence in situ hybridization (FISH) was performed to confirm the karyotype findings. Commercially available DNA probes were used, and analyses were carried out according to the manufacturers’ instructions (Abbott Diagnostics, IL, USA, and MetaSystems, Altlußheim, Germany). Complex karyotype aberrations were confirmed by multicolor FISH, performed as described by the manufacturer (MetaSystems, Altlußheim, Germany).

### 2.5. Statistical Analysis

Patient demographics were summarized using descriptive statistics. Continuous variables were presented as mean ± standard deviation (SD) for normally distributed. Differences between groups were compared using the two-sided Student’s *t*-test or the Mann–Whitney U test, depending on data distribution. Categorical variables were analyzed using the chi-square test. A *p*-value < 0.05 was considered statistically significant. Statistical analyses were performed using SPSS version 23 (IBM Corp., Armonk, NY, USA). Given the limited sample size, these data should be interpreted descriptively.

## 3. Results

### 3.1. Patient Characteristics

In total, 511 patients were analyzed with 63 patients included in this retrospective analysis fitting the inclusion criteria. Patient characteristics including demographic and clinical parameters are shown in Table 1. All patients received cytogenetic analysis at the diagnosis and at relapse after allo-HSCT.

CGE, defined as any form of cytogenetic change (evolution, devolution, or combined patterns), was detected in 29 out of 63 patients (46.1%).

No difference in age at the diagnosis of hematological neoplasia and allo-HSCT was seen between the patient groups with and without CGE, respectively, with an average age 57.1 years for the CGE group and 58.4 years for the non-CGE group. Furthermore, a similar distribution in gender was observed between these two patient groups as well (55.9 vs. 51.7% male and 44.1 vs. 48.3% female, respectively).

The mean time from initial diagnosis to transplantation was 5 months in the CGE group and 4 months in the non-CGE group.

The mean time from allo-HSCT to relapse was 6 months in the CGE group and 9 months in the non-CGE group.

### 3.2. Clinical Correlations

In CGE group, 20 out of 29 patients (69.0%) had AML comparing to the 22 out of 34 (64.7%) in non-CGE group. The prevalence of other hematological malignances was also similar in both patient groups (MDS 20.7% vs. 20.6%, MPN 6.9% vs. 8.8%, and chronic myelomonocytic leukemia (CMML) 3.4% vs. 2.9%, respectively).

Furthermore, we investigated whether prior chemotherapy exposure influenced the development of CGE. A similar proportion of the patients received chemotherapy prior to allo-HSCT in the CGE and non-CGE groups (89.7 vs. 88.2%). Chemotherapy intensity exists along a continuum, ranging from low-intensity regimens, such as hypomethylating agents with or without venetoclax, to intermediate cytoreductive approaches, including hydroxycarbamide and low-dose cytarabine (Ara-C), and extending to high- and very high-intensity induction regimens administered with curative intent. These intensive strategies include combinations such as the “7+3” regimen (cytarabine plus daunorubicin), liposomal daunorubicin–cytarabine (CPX-351/Vyxeos), and high-dose cytarabine-based protocols such as HAM (cytarabine plus mitoxantrone) or FLAG-Ida (Fludarabine, Ara-C, Idarubicin, and G-CSF). No significant difference was observed regarding the type of conditioning regimen: reduced intensity in 58.6 vs. 64.7%, and myeloablative in 41.4% vs. 35.3% in the CGE vs. non-CGE group, respectively.

Moreover, donor type did not differ significantly between the CGE and non-CGE groups. The distribution of matched related donors (MRD), matched unrelated donors (MUD), mismatched unrelated donors (MMUD), and haploidentical donors was comparable in both cohorts. In our study, only five patients received total body irradiation (TBI) as a part of the conditioning regime: one patient in the CGE (3.4%) and four (11.8%) in the non-CGE group. However, the small number of patients did not allow a meaningful statistical analysis.

### 3.3. Cytogenetic Characteristics at the Initial Diagnosis and at the Relapse After Allo-HSCT

**Figure 1:**
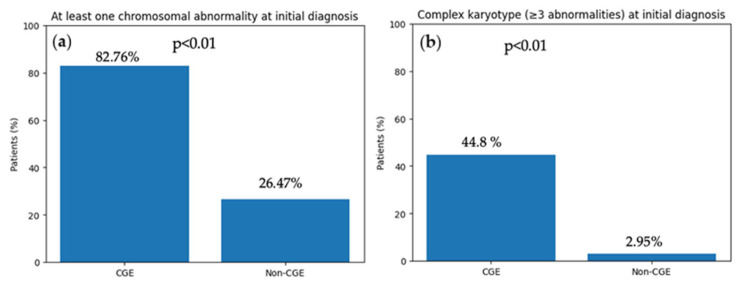
(**a**) **Frequency of chromosomal abnormalities at initial diagnosis in patients with and without cytogenetic evolution (CGE).** The proportion of patients presenting with at least one chromosomal abnormality at initial diagnosis was significantly higher in the CGE group compared with the non-CGE group (23/29 [82.76%] vs. 9/34 [26.47%], respectively; *p* < 0.01). (**b**) **Prevalence of complex karyotype at initial diagnosis in patients with and without cytogenetic evolution (CGE).** A significantly higher proportion of patients in the CGE group exhibited a complex karyotype, defined as three or more chromosomal abnormalities, at initial diagnosis compared with patients in the non-CGE group (13/29 [44.8%] vs. 1/34 [2.95%], respectively, *p* < 0.01).

In total 29 out of 63 patients (46.03%) developed a cytogenetic evolution in our study cohort. A significantly higher number of patients in the CGE group (23 out of 29, 82.76%) had at least one chromosomal change at the initial diagnosis compared to the non-CGE group (9 out of 34, 26.47%) (*p* < 0.01), Figure 1a. Furthermore, a significantly higher number of patients in the CGE group (13 out of 29, 44.8%) had three and more chromosomal changes at the initial diagnosis demonstrating a complex karyotype, comparing to the non-CGE group (1 out of 34, 2.95%, *p* < 0.01), Figure 1b.

In the CGE group, only 7 out of 29 patients (24.14%) patients had a normal karyotype at the initial diagnosis, comparing to 26 out of 34 (76.47%) in the non-CGE group.

**Figure 2:**
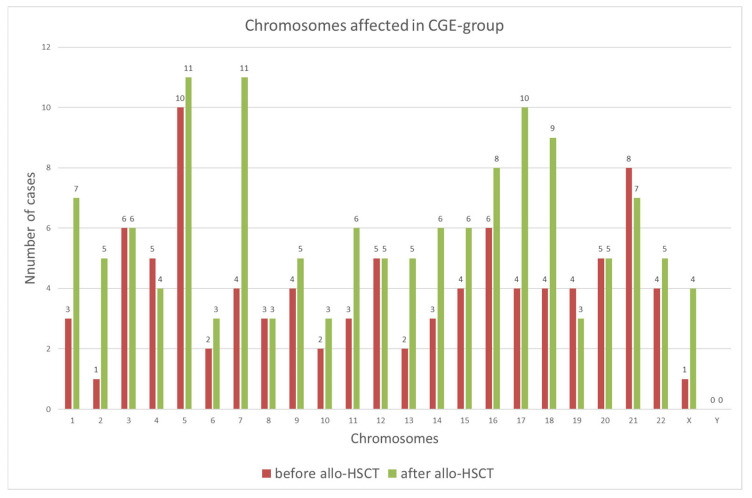
**Chromosomes affected in CGE group at initial diagnosis and after allo-HSCT.** Distribution of chromosomal abnormalities in the CGE group at initial diagnosis and after allogeneic stem cell transplantation (allo-HSCT). The bar chart shows the number of cases with chromosomal involvement for each chromosome at initial diagnosis (red) and at relapse after allo-HSCT (green).

At the initial diagnosis, almost all chromosomes, except chromosome Y, were affected in the CGE group. The most frequently involved chromosomes were chromosome 5 (34.5%) and chromosome 21 (27.6%), followed by chromosomes 3 and 16 (20.7% each) (Figure 2). In contrast, alterations of chromosomes 2 and X were rare, each observed in only one case (3.4%). All remaining chromosomal abnormalities occurred at intermediate frequencies and are summarized in Figure 2.

At relapse, the frequency of chromosomal alterations increased across nearly all chromosomes, again with the exception of chromosome Y (Figure 2). Chromosomes 5 and 7 were most frequently affected (each 37.9%), followed by chromosome 17 (34.5%). Conversely, chromosomes 6, 10, and 19 were among the least frequently involved (each 10.3%). Notably, chromosomes 3 and 8 showed no appreciable differences in alteration frequency compared with the non-relapsed samples. The detailed distribution of all remaining chromosomal abnormalities at the initial diagnosis and after allo-HSCT is shown in Figure 2.

Chromosomes affected in the non-CGE group are chromosome 3 in 2 out of 34 cases (5.9%), 7 and 8 in 3 out of 34 (8.8%), and 9, 12, 16, 20 and 22 in 1 out of 34 (2.9%) cases, each.

Table S1 summarizes the cytogenetic findings at initial diagnosis and at relapse after allo-HSCT in patients who developed karyotypic evolution, whereas corresponding data for patients without karyotypic evolution are presented in Table S2.

Detailed information on patients with AML, with or without karyotype evolution, is provided in Tables S3 and S4.

Chromosomal alterations in the CGE group did not follow a uniform or recurrent pattern but were predominantly characterized by the acquisition of new chromosomal abnormalities or the emergence of novel clones.

Karyotypic changes most frequently consisted of clonal evolution with the addition of two or more new chromosomal abnormalities (15 of 29 cases, 51.7%), followed by the acquisition of a single new chromosomal abnormality (6 of 29 cases, 20.7%).

In contrast, clonal devolution, defined as the loss of previously detected chromosomal abnormalities, was less common and occurred as the loss of one abnormality in three of 29 cases (10.3%) and the loss of two or more abnormalities in four of 29 cases (13.8%). One patient (3.5%) demonstrated a mixed pattern, with the loss of one pre-existing abnormality accompanied by the acquisition of multiple new chromosomal changes. The distribution of clonal evolution patterns in patients with CGE group at relapse after allo-HSCT is shown in Figure S1.

### 3.4. Association of CGE with Survival

An association between day 30 response after allo-HSCT and later-defined CGE status was observed. Complete remission rates at day 30 were 48.3% in the CGE group and 82.4% in the non-CGE group (*p* = 0.001). However, this finding should be interpreted with caution and cannot be considered predictive of cytogenetic evolution. Rather, it may reflect shared underlying disease aggressiveness.

**Figure 3:**
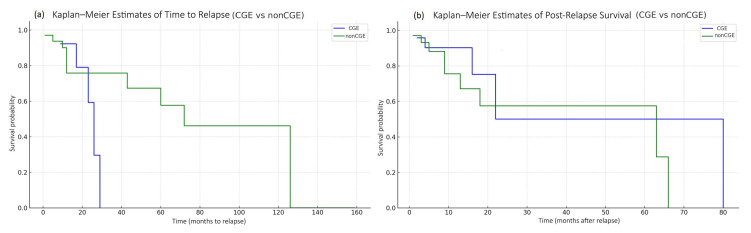
(**a**) Kaplan–Meier analysis of time to relapse after allo-HSCT showed no statistically significant difference between patients with and without cytogenetic evolution (CGE) (log-rank *p* = log-rank *p* = 0.679). (**b**) Kaplan–Meier analysis of post-relapse survival demonstrated no statistically significant difference between the CGE and non-CGE groups (log-rank *p* = 0.394) as well.

Kaplan–Meier analysis of time to relapse after allo-HSCT showed no statistically significant difference between patients with and without CGE (log-rank *p* = 0.679) (Figure 3a).

Similarly, Kaplan–Meier analysis of post-relapse survival demonstrated no statistically significant difference between the CGE and non-CGE groups (log-rank *p* = 0.394) (Figure 3b).

Given the small sample size, sparse subgroup counts, and the exploratory nature of the unadjusted comparisons, these findings should be interpreted with caution.

## 4. Discussion

Relapse remains the leading cause of treatment failure in patients with myeloid malignancies following allogeneic hematopoietic cell transplantation [22,23,24,25,26,27,30,31,32]. Although the biological mechanisms underlying post-transplant relapse and therapy resistance are incompletely understood, increased evidence suggests that clonal cytogenetic changes, including the acquisition or loss of chromosomal abnormalities may play an important role [9,11,16]. Accordingly, karyotypic alterations represent not only markers of genomic instability but also potential prognostic determinants of relapse in patients treated with intensive chemotherapy or allo-HSCT [11,15,17,33].

Cytogenetic changes in the form of karyotype evolution are a well-recognized phenomenon in relapsed myeloid malignancies, occurring both after conventional chemotherapy and following allo-HSCT [7,8,9,10,11,12,13,14,15,16,17]. However, its determinants and clinical implications in the transplant setting remain incompletely defined. In particular, data are limited regarding the potential association between specific types of chemotherapy, conditioning intensity, or donor characteristics and the development of cytogenetic evolution at relapse [9,11,16]. Moreover, the impact of CGE on post-relapse survival in patients undergoing allo-HSCT has not been sufficiently characterized.

In this retrospective analysis of 63 patients with myeloid hematologic neoplasms relapsing after allo-HSCT, cytogenetic changes were observed in 46.1% of cases.

This frequency is in line with previously published data reporting rates of cytogenetic changes between 51% and 68% [9,11,16]. The slightly lower incidence observed in our cohort is likely attributable to differences in study design and patient composition.

In contrast to prior reports by Bacher et al. and Schmidt-Hieber et al. [11,16], which exclusively analyzed patients with acute myeloid leukemia, our study included a broader spectrum of myeloid hematologic neoplasms.

Given the biological and cytogenetic heterogeneity across these entities, inclusion of non-AML diagnoses may dilute the overall rate of detectable cytogenetic evolution, thereby providing a more comprehensive but less disease-restricted estimate.

In contrast to the study by Schmidt-Hieber et al. [16], who reported a significantly younger age in patients with cytogenetic evolution, we did not observe age-related differences between the CGE and non-CGE groups in the present study. This discrepancy likely reflects differences in cohort size and disease restriction, as their analysis was limited to a small AML-only population. In line with our findings, Ertz-Archambault et al. [9] likewise reported comparable ages across groups, suggesting that age is unlikely to represent a major determinant of cytogenetic evolution.

Importantly, we did not identify an association between prior chemotherapy exposure and the development of cytogenetic evolution The proportion of patients receiving chemotherapy before allo-HSCT was comparable between the CGE and non-CGE groups, and neither the intensity of conditioning regimens nor donor type appeared to influence the occurrence of CGE. These findings suggest that treatment-related factors alone may not fully explain the emergence of cytogenetic evolution following transplantation.

Our results are highly consistent with those reported by Ertz-Archambault et al. [9], who performed a detailed analysis of induction chemotherapy, lines of salvage therapy, and pretransplantation conditioning regimens and likewise found no significant association with CGE. Despite substantial heterogeneity in both induction and salvage regimens, the cumulative number of pre-allo-HSCT treatment lines and the spectrum of conditioning therapies were comparable between the CGE and non-CGE cohorts in their study. Together with our data, these findings argue against the dominant role of chemotherapy intensity or regimen selection in driving cytogenetic evolution.

In our cohort, cytogenetic evolution was strongly associated with the presence and complexity of cytogenetic abnormalities at the initial diagnosis. Patients who later developed CGE were significantly more likely to harbor at least one chromosomal aberration at baseline, and a complex karyotype was markedly enriched in this group, whereas a normal karyotype at diagnosis was predominantly observed in patients without subsequent cytogenetic evolution. These findings support the concept that pre-existing cytogenetic instability predisposes to clonal evolution following allo-HSCT.

Our results are consistent with those reported by Ertz-Archambault et al. [9], who likewise observed a higher frequency of abnormal and complex karyotypes at baseline in patients with CGE within a diagnostically heterogeneous myeloid cohort. Importantly, similar observations have been described in acute myeloid leukemia outside the transplant setting, where karyotype evolution at relapse occurred more frequently in patients with unfavorable cytogenetic profiles at diagnosis [17]. In that study, the acquisition of additional chromosomal aberrations was significantly more common in patients with adverse-risk karyotypes compared with other cytogenetic risk groups, underscoring baseline karyotype instability as a critical determinant of subsequent clonal evolution.

Collectively, these data suggest that increasing cytogenetic complexity at initial diagnosis may be associated with a higher risk of clonal evolution at relapse. Rather than being primarily driven by transplant-related or therapy-induced mutagenesis, our findings suggest that cytogenetic evolution may, at least in part, reflect intrinsic genomic instability of the leukemic clone, which becomes further unmasked under therapeutic pressure. Thus, baseline karyotype complexity may represent a key biological determinant of genomic dynamics in myeloid neoplasms following allo-HSCT.

Analysis of chromosomal involvement in our cohort demonstrated non-random patterns of cytogenetic evolution, supporting the concept of biologically driven clonal selection rather than stochastic chromosomal damage. At the initial diagnosis, nearly all chromosomes, except chromosome Y, were already involved in patients who later developed cytogenetic evolution (CGE), with chromosomes 5 and 21 being the most frequently affected. At relapse, the overall burden of chromosomal alterations increased further, most prominently involving chromosomes 5, 7, and 17. These chromosomes are well recognized for their association with adverse-risk disease biology and genomic instability in myeloid neoplasms [14,34,35,36,37].

Our findings align with previous observations that specific chromosomal abnormalities, particularly monosomy 7 or deletion 7q, are frequently acquired during disease progression and are associated with inferior outcomes. Jabbour et al. reported that chromosome 7 alterations represented the most common newly acquired abnormality (21% of cases with acquired cytogenetic abnormalities) in lower-risk MDS and were strongly linked to leukemic transformation [13]. Similarly, Schanz et al. and Valcárcel et al. identified −7/del(7q) and del(17p) as particularly unfavorable within cytogenetic evolution patterns in MDS, underscoring the biological relevance of these high-risk chromosomes [14,38].

However, data specifically addressing relapse after allogeneic hematopoietic cell transplantation (allo-HSCT) present a more heterogeneous picture. Schmidt-Hieber et al. [16], demonstrated a non-random distribution of newly acquired structural abnormalities after allo-HSCT but did not identify a single dominant relapse-defining chromosome.

Similarly, Ertz-Archambault et al. [9] did not observe consistent recurrent cytogenetic patterns at relapse after allo-HSCT. Importantly, deletions of chromosomes 5/5q, 7/7q, and 11q23 translocations were detected in several patients; however, these abnormalities were already present at diagnosis and therefore did not represent newly acquired relapse-specific events. Their data suggest that post-transplant relapse does not necessarily follow a uniform cytogenetic trajectory dominated by a single chromosome but rather reflects diverse clonal dynamics influenced by prior therapy and transplant-related selective pressures.

Taken together, these observations indicate that while certain chromosomes—particularly 5, 7, and 17—are consistently associated with adverse biology and frequently involved in cytogenetic evolution across myeloid malignancies, the post-transplant relapse setting may not be characterized by a single, universally dominant chromosomal lesion. Instead, cytogenetic evolution after allo-HSCT appears to reflect structured but heterogeneous trajectories shaped by intrinsic genomic instability, pre-existing subclones, prior chemotherapy, and transplant-related selection mechanisms.

Cytogenetic evolution was associated with an inferior early post-transplant response, as reflected by lower complete remission rates on day 30 in patients with CGE. However, despite this early disadvantage, neither relapse-free survival nor post-relapse survival differed significantly between the two groups in our analysis.

Published data on the prognostic impact of CGE after allo-HSCT remain heterogeneous. While Ertz-Archambault et al. [9] reported inferior post-transplant and post-relapse survival in patients with CGE, Schmidt-Hieber et al. [16] did not observe significant survival differences. Notably, these discrepancies likely reflect variations in cohort size, disease composition, and post-relapse management.

Platte et al. [21] and Yeung et al. [22] support the notion that disease biology, as reflected by cytogenetics, is a key factor influencing both the timing of relapse and the response to relapse treatment.

Our findings align in part with both reports, demonstrating a clear association between CGE and impaired early post-transplant response, while failing to confirm a statistically significant impact on long-term survival endpoints. Collectively, these observations suggest that CGE may primarily capture early disease aggressiveness rather than independently determining long-term survival outcomes.

Taken together, our findings support the hypothesis that relapse after allo-HSCT may be driven, at least in part, by the expansion of biologically advantaged pre-existing subclones rather than by a uniform transplant-associated cytogenetic event. However, several limitations should be considered. The retrospective single-center design, limited sample size, and absence of multivariable analyses restrict the statistical power and generalizability of the findings, and potential confounding factors cannot be fully excluded. In addition, the analysis was limited to patients who relapsed after allo-HSCT and did not include a non-relapse comparison cohort, which may introduce selection bias. Although AML represents the majority of cases, subgroup analyses restricted to AML were not performed due to limited sample size, as such analyses would be underpowered and potentially misleading. Furthermore, molecular profiling data were not incorporated, as standardized MRD-relevant markers are currently limited, which may restrict the biological interpretation of clonal evolution. Therefore, these findings should be interpreted as exploratory and hypothesis-generating. Larger, disease-specific studies integrating cytogenetic and molecular data are warranted to further clarify the underlying mechanisms and clinical relevance of clonal evolution in this setting.

## 5. Conclusions

In this retrospective analysis of patients with myeloid hematologic neoplasms relapsing after allogeneic hematopoietic cell transplantation, cytogenetic evolution occurred in nearly half of the cases and was strongly associated with baseline cytogenetic complexity. Treatment-related variables, including prior chemotherapy exposure, conditioning intensity, and donor type, did not significantly influence the development of cytogenetic evolution.

These findings raise the possibility that post-transplant cytogenetic evolution may be related to underlying genomic instability and clonal selection, while the contribution of therapy-induced mutagenesis remains to be clarified.

While cytogenetic evolution was associated with impaired early post-transplant response, its impact on long-term survival remains uncertain. Larger, disease-stratified studies integrating cytogenetic and molecular longitudinal analyses are required to further define the biological and prognostic implications of clonal evolution following allo-HSCT.

## Figures and Tables

**Table 1:** Demographic, clinical and laboratory patient characteristic.

*n*	With Cytogenetic Clonal Evolution *n* = 29	Without Cytogenetic Clonal Evolution *n* = 34	*p*-Value
Age (years) at diagnosis of neoplasia, (mean ± SD)	57.07 (12.820)	58.41 (12.863)	0.827 **
Age (years) at allo-HSCT, (mean ± SD)	58.79 (12.190)	58.41 (12.863)	0.906 **
Time to relapse after allo-HSCT (months, mean ± SD)	18.03 (35.027)	29.00 (40.789)	0.106 **
Time (months) after relapse, (mean ± SD)	10.59 (18.076)	12.97 (17.728)	0.643 **
Gender, *n* (%)			
male	15 (51.7)	19 (55.9)	0.939 ***
female	14 (48.3)	15 (44.1)	
Underlying malignancy, *n* (%)			
AML	20 (69.0)	22 (64.7)	0.913 ***
MDS	6 (20.7)	7 (20.6)	
MPN	2 (6.9)	3 (8.82)	
CMML	1 (3.4)	1 (2.94)	
Other °	0 (0)	1 (2.94)	
Chemotherapy before allo-HSCT, *n* (%)			
yes	26 (89.7)	30 (88.2)	0.510 ***
no	3 (10.3)	4 (11.8)	
Response before allo-HSCT, *n* (%)			
CR *	9 (31.02)	8 (23.53)	0.001 *****
SD	6 (20.7)	5 (14.7)	
PR or MRD pos °	7 (24.14)	13 (38.24)	
nonresponse	7 (24.14)	8 (23.53)	
Response after allo-HSCT on day 30, *n* (%)			
CR *	14 (48.3)	28 (82.4)	0.02 ***
SD	4 (13.8)	0	
PD or MRD pos °	5 (17.2)	6 (17.6)	
nonresponse	6 (20.7)	0	
Conditioning regimen prior to allo-HSCT, *n* (%)			
reduced intensity	17 (58.6)	22 (64.7)	0.814 ***
myeloablative	12 (41.4)	12 (35.3)	
non-myeloablative	0	0	
total body irradiation	1 (3.4)	4 (11.8)	
Donor type, *n* (%)			
MRD	5 (17.24)	2 (5.9)	0.136 ***
MUD	15 (51.72)	27 (79.4)	
MMUD	6 (20.69)	3 (8.8)	
Haploidentical	3 (10.35)	2 (5.9)	
Number of cytogenetic changes at initial diagnosis, *n* (%)			
0	6 (20.69)	25 (73.53)	<0.01 ***
≥1	23 (79.31)	9 (26.47)	
≥3	13 (44.8%)	1 (2.95%)	<0.01 ***
Survival Status			
Alive	5 (17.24)	9 (26.47)	Time to relapse after allogeneic transplantation 0.679 ****
Dead	24 (82.76)	24 (70.59)	
unknown	0 (0)	1 (2.94)	Time after relapse 0.394 ****

Allo-HSCT, allogeneic hematopoietic stem cell transplantation; AML, acute myeloid leukemia; CMML, chronic myelomonocytic leukemia; CR, complete remission; MMUD, mismatched unrelated donor; MPN, myeloproliferative neoplasia; MDS, myelodysplastic neoplasia; MRD, matched related donor MUD, matched unrelated donor; PR, partial remission; PD, progressive disease; SD, stable disease, * CR is confirmed in flow cytometry and NGS; ° MRD positive is confirmed in flow and/or NGS. ** Student *t*-test; *** χ^2^ test; **** Kaplan–Meier test; ***** Due to low expected cell counts and zero cells in the contingency table, the association between response on day 30 after allo-HSCT and CGE/noCGE status was analyzed using Fisher’s exact test for a 2 × 4 contingency table. The association remained statistically significant (*p* = 0.001).

## Data Availability

The datasets used and/or analyzed during this study are available from the corresponding author upon reasonable request.

## Supplementary Materials

The following supporting information can be downloaded at: https://www.mdpi.com/article/10.3390/cancers18101665/s1, Figure S1: Distribution of cytogenetic change patterns in the CGE group at relapse after allo-HSCT; Table S1: Diagnosis, karyotype at initial diagnosis and at relapse after allo-HSCT in patients developing a karyotype evolution; Table S2: Diagnosis, karyotype at initial diagnosis and at relapse after allo-HSCT in patients not developing a karyotype evolution; Table S3. Analysis of patient data in AML with developing a karyotype evolution; Table S4. Analysis of patient data in AML without developing a karyotype evolution.

## Abbreviations

The following abbreviations are used in this manuscript:

AML	acute myelogenous leukemia
allo-HSCT	allogeneic hematopoietic stem cell transplantation
Ara-C	Cytarabine
CGE	cytogenetic clonal evolution
CML	chronic myelocytic leukemia
CMML	chronic myelomonocytic leukemia
CPX-351/Vyxeos	liposomal daunorubicin and cytarabine
CR	complete remission
DIPSS	dynamic international prognostic scoring system
ELN	European leukemia network
EPO	erythropoietin
ET	essential thrombocythemia
FISH	fluorescence in situ hybridization
FLAG-Ida	fludarabine, Ara-C, G-CSF (Filgrastim), and Idarubicin
G-CSF	granulocyte colony-stimulating factor
HAM	high-dose cytarabine and mitoxantrone
IPSS-R	Revised International Prognostic Scoring System
IQR	interquartile range
ISCN	international system for human cytogenomic nomenclature
MDS	myelodysplastic syndromes
MDS-MLD	myelodysplastic syndrome with multilineage dysplasia
MMUD	mismatched unrelated donors
MPAL	mixed phenotype acute leukemia
MPN	myeloproliferative neoplasms
MRD	matched related donors
MRD	minimal residual disease
MUD	matched unrelated donors
NGS	next generation sequencing
non-CGE	without cytogenetic clonal evolution
PD	progressive disease
PMF	primary myelofibrosis
PR	partial remission
PV	polycythemia vera
sAML	secondary acute myeloid leukemia
S-HAM	sequential high-dose cytarabine and mitoxantrone
SD	stabile disease
SD	standard deviation
tAML	therapy-related acute myeloid leukemia
TAD9	Thioguanine, Ara-C, Daunorubicin
TBI	total body irradiation
TKI	tyrosine kinase inhibitors
WHO	World Health Organization
7+3	cytarabine plus daunorubicin
