# Supplementary material for: Clonal Cytogenetic Evolution in Relapse of Myeloid Hematological Neoplasms After Allogeneic Stem Cell Transplantation
Source: Cancers (Basel). 2026 May 21;18(10):1665. doi: 10.3390/cancers18101665 (PMC13204579; doi:10.3390/cancers18101665)
Supplement: Supplementary file 1 [file cancers-18-01665-s001.zip › cancers-4245776-supplementary.pdf]

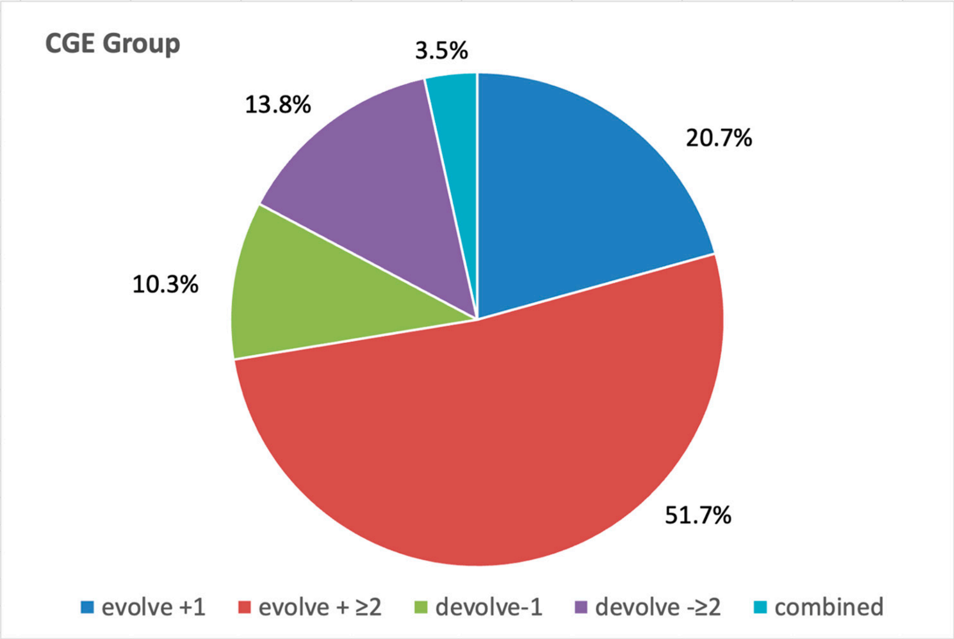

Figure S1. Distribution of cytogenetic change patterns in the CGE group at relapse after allo-HSCT. The majority of cases were characterized by the acquisition of two or more new chromosomal abnormalities (51.7%), followed by the addition of a single new abnormality (20.7%). Clonal devolution with loss of one (10.3%) or two or more (13.8%) previously detected abnormalities was less frequent. A minority of patients (3.5%) demonstrated a combined pattern, with simultaneous loss of pre-existing abnormalities and acquisition of new chromosomal changes.

Table S1. Diagnosis, karyotype at initial diagnosis and at relapse after allo-HSCT in patients developing a karyotype evolution.

Nr.	Gender/age at allo-HSCT	Disease/risk stratification	Karyotype at initial diagnosis	Karyotype at relapse after allo-HSCT
1	m /50	AML, ° ELN high risk	46,XY,del(7)(q32q36)[5]/46,XY[15]	46,XX[15]/46,XY [5]
2	w/55	AML, ELN intermediate risk	47,XX,+8[19],46,XX[1]	46,XX,t(2;10;14)(p23;q23;q11),t(10;12)(q24;q24),der(14)t(14;17)(q32;q22),r(18)(p11q12)[13]/46,XX[7]
3	m/66	AML, ELN high risk	46,XY,t(1;3)(p3?6;q26),t(9;22)(q34;q11.2)[3]/55,idem,+Y,+8,+10,+12,+15,+19,+20,+21,+der(22)t(9;22)(q34;q11.2)[2]	44,XY,t(1;3)(p3?6;q26),-7,der(9)t(9;22)(q34;q11.2),-20,der(22)t(9;22)(q34;q11.2)t(9;20)(q34;q1?1)[22]
4	w/59	AML, ELN high risk	45 XX,-7[19]/46,XX[1]	45,XX,-7[11]/46,XX,+i(X)(p10),-7[4]/46,XY[12]
5	w/72	AML with MDS-related changes, ELN high risk	44,XX,der(4)del(4)(p12)t(4;17)(q21;q12), der(5)t(5;16)(?p12;p12)t(5;16)(q1?4;q21)t(16;9)(q2?4;q3?3),-7,der(9)t(7;9)(?p?;q3?3)+11,-16,-17,-18,der(20)t(4;20)(?q?;q1?3)t(4;7)(?q?;q?31),+21[18]/46,XX[5]	43,XX, der(4)del(4)(p12)t(4;17)(q21;q12), der(5)t(5;16)(?p12;p12)t(5;16)(q1?4;q21)t(16;9)(q2?4;q3?3), der(9)t(7;9)(?p?;q3?3)t(7;20),+11,-16,-17,-18,der(19)ins(19;21)(q13;q11.2q22),del(19)(p12), der(20)t(4;20)(?q?;q1?3)t(4;7)(?q?;q?31)[21]
6	m/59	Post PV myelofibrosis, DIPSS * intermediate II	46,XY,+1,der(1;18)(q10;q10)[6]/47,idem,+9 [2]/47,idem,+del (9)(q22q34)[3]/49,idem,+6,+9,+21[6]/46,XY[4]	46,XY,+1,der(1;18)(q10;q10),+9,-11[3]/47,idem,+del(9)(q22q34)[17]

7	w/58	AML, ELN high risk	46,XX[25]	46,XX, der(1)(p22p36)t(1;15)(q21;q2?3),-5,der(13)t(1;13)(p31;q3?3), der(17)t(3;17)(q2?1;p12),+mar[3]/46,XX[12]
8	w/60	AML, ELN intermediate risk	46,XX[10]	45,XX,t(1;2)(p21;q36), der(2)t(2;18)(q22;q11),inv(3)(p12q28),inv(7)(p14q35), -18,der(20)t(18;20)(?p11;q13)[9]/ 46,XX,t(15;15)(q26;q11),ins(16;11)(q2?1;q13q24)[9]/46,XX,inv(3)(p21q27)[3]
9	w/64	MDS, IPSS-R ** intermediate risk	46,XY,+8[16]/46,XX [4]	47,XX,+8 [17]/49XX,der(7)t(7:17)(p22;q22),+8,+8,+21 [2]/50XX, der(7)t(7;17)(p22;q22),+8,+8,+13,+21 [4]/46,XX[1]
10	m/50	sAML from MDS ELN high risk	45,XY,-5,-14,-16,add(17)(p11.2),-18,del(20)(q11,2) or-20,+22,+r(?),+mar1,+mar2 [1]/46,XY,idem,-13, +mar3 [25]/46,XY[1]	43-45,XY,t(5;?),-14,-16,add(17)(q11.2),-18,del(20)(q12)or -20,+22,+mar1, +mar2,+dmin20]
11	w/29	AML, ELN good risk, relapse	46, XX,inv(16)(p13q22)[15]	46,XX,inv(16)(p13q22)[15]/46XX,del(7)(q22q36),inv(16)(p13q22)[4]/46,XY[3]
12	m/75	AML, ELN high risk	46,XY,del(5)(q14q34),r(7)(p11q11),i(8)(q10),ins(9;14)(p24;q22q32),der(16;21)(p10;q10),-18, +21+r(21)(p11q22)[11]	43-45,Y,(X)(15qter-> 15q25::Xp21::15q13->15q25::Xq27->Xqter), del(5)(q14q34),-7,ins(9;14)(p24;q22q32),r(15)(p11q13), der(16;21)(p10;q10),r(17;21)(p13p10;q10q22),-18, +21, +der(21)t(17;21)(q11;22),+r(21)(p11q22)[cp2]/46,XX [15]
13	w/26	MDS, IPSS-R intermediate risk	46,XX[20]	48,XX,+X,+8[5],46,XX[16]
14	w/70	AML, ELN high risk	45,X,-X,der(3)t(3;16)(p21;p11), der(5)t(5;10)(q14;p12),-7, der(10)t(5;10)(q34;p12),der(12)t(3;12)(q21;q13),del(14)(q21q31),-16, der(20;22)(q10;q10)x3[12]/ 90, XX,-X,-X, der(3)t(3;16)(p21;p11)x2,der(5)t(5;10)(q14;p12)x2,-7,-7, der(10)t(5;10)(q34;p12)x2,-12,-12,der(12)t(3;12)(q21;q13), del(14)(q21q31)x2,-16,-16,der(20;22)(q10;q10)x2, +der(20;22)(q10;q10)x6[3]/46,XX[4]	42,X,-X,der(1)t(1;9)(q44;q22)t(4;9)(q?27;q34) t(4;5)(q34;q34),der(3)t(3;16)(p21;p11)del(3)(q12q29),-4,der(5)t(5;10)(q14;p12),-7,der(9)ins(9;3)(q13;?p24?p26)del(9)(q22q34), der(10)t(X;10)(?p21;p12),der(12)t(3;12)(q21;q13),der(12)t(4;12)(?p13;p11), del(14)(q21q31),-16 [8]/46,XX[3]
15	m/46	tAML, ELN intermediate risk	46,XY,t(8;16)(p11;p13)[15]/46,XY[5]	46,XY,t(8;16)(p11;p13) [1]/46,idem,t(4;18)(q31;q22)[8]/46,XY[11]
16	m/50	PMF, DIPSS * high risk	46,XY[21]	47,XY,t(1;11)(p32;q23),der(6;8)(q10;q10),+8,der(8) t(6;8)(p2?1;q12),+i(8)(q10)[5]/ 48,XY,t(1;11)(p32;q23),der(6;8)(q10;q10),+der(6;8)(q10;q10),+8, der(8)t(6,8)(p2?1;p12),+i(8)(q10)[5]/46,XY[10]

17	m/65	MDS, IPSS-R very high risk	47,XY,der(1)t(1;5)(p21;p11),+der(1)t(1;5)(p21;p11),der(2)ins(2;1)(q32;??),-5,+8, der(15)t(1;15)(p34;q22)[9]/47,idem,+8 [2]/46,XY[9]	46,XY,der(1)t(1;5)(p21;p11),+der(1)t(1;5)(p21;p11),der(2)ins(2;1)(q32;??),-5, der(15)t(1;15)(p34;q22)[7]/46,XY[13]
18	m/65	MDS, IPSS-R very high risk	45,XY,del(5)(q31),-7[20]	44, XY,-2,der(2)t(2;14)(p2?3;q12),t(3;21)(q2?5;q21),-5,der(5;20)(p10;q10), t(6;18)(p2?2;q11.2),der(7)t(7;?19)(q21;?),-14,der(22)t(5;22)(q3?3;p12), del(18)(q11.2),del(19)(?q13),+mar,+mar[10cp]/46,XY[14]
19	m/68	AML, ELN high risk	44,XY,-4,-5,-15,-16,-19,-21,+6mar,3dmins[10]	46-50,XY, del(4)(q31q35),+der(5;17)(p10;q10)t(17;21)(q24;q21), der(5;17)(p10;q10), r(10)(p11q11),der(15)(15qter->15q24::15q11->15q26::10q25->10qter),r(16)(p12q12),-19,del(21)(q22q22)t(16;19)(q22;p11),+i(21)(q10),2-4dmin[cp18]/46,XY[4]
20	w/57	MDS, IPSS-R very high risk	44,XX,-5,del(7)(p13),i(7)(p10),-11,-12,add(12)(p11.2),-13,-17,+r,+2mar[8]	45,XX,del(5)(q11.2),del(7)(p13),del(7)(q22),der(12)t(12;12)(p13;q13),add(13)(p11),-17,i(22)(q10),+mar[5]/45,XX,idem,add(15)(p11)[6]/46,XY[2]
21	m/46	AML, ELN high risk	47,XY,+8 [18] /46,XY[2]	47,XY,+8 [18] /47,XY,+8,t(3;7)(p25;q11)[4]
22	m/62	AML, ELN high risk	46,XY[20]	46,XY,-6,del(7)(p13),t(12;14)(q24.1;q22),der(13)t(13;13)(q12;q34),+2mar [10]/46,XY[10]
23	w/70	CMML intermediate II risk	46,XX[20]	46,XX,del(7)(q22q35),t(21;21)(q11.2;q22)[21]/46,XX[1]
24	m/71	MDS-MLD, IPSS-R very high risk	44-46,XY,del(3)(p14p24),del(4)(q22q34), der(5)t(4;5)(q?;q14),der(12)r(12;19)(p12q21;p13.1p13.3), der(15)t(15;18)(p11;q12),dic(17;20)(p12;p13),-18,der(19)t(12;19)(q21;p13),+21,+21,i(21)q10)[cp16]/46,XY[4]	46,XY[8]
25	w/62	AML, ELN high risk	46,XX,del(3)(q13),del(5)(q13),-11,add(21)(q22),-22,+r,+mar,2-3dmin[18]	46,XX,del(3)(q13),del(5)(q13),-11,add(21)(q22),-22,+r,+mar, 2-3dmin[8]/47,XX,idem,+mar[8]
26	w/68	tAML, ELN high risk	45,XX,der(3)t(3;13)(q21;q31),t(5;6)(p15;p21),i(5)(p10),-7,der(13)t(3;13)(q21;q31),inv(q21;q26)[12]	46, XY[20]
27	m/43	sAML from MPN, ELN: intermediate risk	46,XY,t(12:17)(q15;23)[20]	46,XY,del(2)(q11q37),der(11)t(2;11)(q11;p15)del(2)(q13q37),t(12;17)(q15;q23), der(13)t(13;15)(q14;q13),der(15)t(2;15)(q22;q15)t(2;13)(q32;q14)[3]<br>46,XY [1]

28	w/75	AML, ELN high risk	46,XX[20]	47,XX,del(5)(q14q33),add(17)(p13),+mar[5]/46,XY[16]
29	m/60	AML, ELN high risk	50,XY,+4,+5,+8,+20[11]/46,XY[6]	46,XY[10]

Allo-HSCT, allogeneic hematopoietic stem cell transplantation; AML, acute myeloid leukemia; sAML, secondary acute myeloid leukemia; tAML, therapy-related acute myeloid leukemia; CMML, chronic myelomonocytic leukemia ; ELN, European leukemia network; ET, essential thrombocythemia; IPSS-R, Revised International Prognostic Scoring System; MDS-MLD, myelodysplastic syndrome with multilineage dysplasia; PMF, primary myelofibrosis; PV, polycythemia vera; DIPSS, Dynamic International Prognostic Scoring System. * DIPSS score was estimated according to the Passamonti et al. [24]. °ELN criteria were estimated according to European leukemia network 2017 and 2022 [1,25]. ** IPSS-R score was estimated according to Greenberg et al. [26].

Table S2. Diagnosis, karyotype at initial diagnosis and at relapse after allo-HSCT in patients not developing a karyotype evolution.

Nr	Gender/age at allo-HSCT	Disease/risk stratification	Karyotype at initial diagnosis	Karyotype at relapse after allo-HSCT
1	m/65	MDS, IPSS-R * intermediate I	46,XX[9]	46,XX[9]/46,XY[11]
2	w/63	MDS, IPSS-R high	46,XX[20]	46,XX[6]/46,XY[6]
3	m/50	MDS, IPSS-R intermediate II	47,XY,+8[16]	47,XY,+8,[17]/46,XX[3]
4	w/66	AML, ° ELN Intermediate	46,XX[20]	46,XX[20]
5	w/48	AML, ELN good risk, relapse	46 XX[20]	46,XX[20]
6	m/49	MDS, IPSS-R very high risk	46,XY[20]	46,XY[20]
7	m/67	AML with MDS-related changes ELN high risk	45,XY,-7[20]	45,XY,-7[12]
8	w/56	AML, ELN intermediate risk	46,XX[22]	46,XY[20]
9	m/30	MPAL, high risk	46,XY[20]	46,XX[21]
10	w/66	AML, ELN intermediate risk	46,XX[21]	46,XX[6]
11	m/54	sAML from CMML, ELN intermediate risk	47,XY,+8[2]/46,XY[19]	47,XY,+8[15]/46,XY[5]
12	m/48	PMF, DIPSS ** Intermediate II	46,XY[22]	46,XY[22]
13	m/64	sAML from CMML ELN intermediate risk	46,XY[23]	46,XX[20]
14	w/62	sAML from CMML ELN high risk	46,XX[20]	46,XX[16]
15	m/60	Post PV myolofibrosis, DIPSS Intermediate I	46,XY[20]	46,XY [19]/46,XX[2]
16	m/54	AML, ELN low risk, relapse	46,XY[20]	46,XY [20]
17	w/75	sAML from MDS, ELN intermediate risk	46,XX[20]	46,XX[20]
18	w/22	AML, ELN high risk	46,XX[13]	46,XY[20]
19	m/66	CMML intermediate 1 risk	46,XY[20]	46,XY[26]
20	w/59	Post PV myolofibrosis, DIPSS Intermediate II	46,XX[20]	46,XY[10]
21	w/56	AML, ELN Intermediate risk	46,XX[20]	46,XY [20]
22	w/70	MDS, IPSS-R high	46,XX[20]	46,XX[20]
23	m/62	myeloid blast phase CML	46,XY,t(9;22)(q34;q12.2)[2]	46,XY,t(9;22)(q34;q12.2)[2]/46,XY[18]
24	m/62	MDS, IPSS-R intermediate	46,XY[20]	46,XY[20]

25	m/60	sAML from CMML ELN high risk	48XY,+8,+8,der(12)t(3;12)(q21;p13) [2]/46,XY[20]	48XY,+8,+8,der(12)t(3;12)(q21;p13)[2]/46,XY[10]
26	w/26	AML, ELN high risk	45,XX,inv(3)(q21q26),-7[20]	45,XX,inv(3)(q21q26),-7[16]/46,XY[4]
27	m/62	AML, ELN good risk, relapse	46,XY,inv(16)(p13q22)[20]	46,XY,inv(16)(p13q22)[2]/46,XY[18]
28	m/71	sAML from ET	46,XY,del(20)(q11q13)[15]/46,XY[2]	46,XY,del(20)(q11q13)[2]/46,XY[18]
29	w/60	sAML from MDS ELN Intermediate risk	46 XX[22]	46,XX[4]/46,XY[16]
30	m/47	sAML from MDS ELN high risk	45 XY,-7[10]	45,XY,-7[3]
31	w/76	AML, ELN Intermediate risk	46,XX [20]	46,XX [20]
32	m/67	sAML from CMML	46,XY [20]	46,XY[3]/46,XX[17]
33	73/w	MDS, IPSS-R: High	46,XX[20]	46,XX[3]
34	68/m	sAML from MDS, ELN: intermediate risk	46,XX[6]	46,XX[7]

Allo-HSCT, allogeneic hematopoietic stem cell transplantation; AML, acute myeloid leukemia; sAML, secondary acute myeloid leukemia; CML, chronic myelocytic leukemia ; ELN, European leukemia network; ET, essential thrombocythemia; IPSS-R, Revised International Prognostic Scoring System; MDS-MLD, myelodysplastic syndrome with multilineage dysplasia; MPAL, mixed phenotype acute leukemia; PMF, primary myelofibrosis; PV, polycythemia vera; DIPSS, Dynamic International Prognostic Scoring System. * IPSS-R score is estimated according to Greenberg et al. [26]. ** DIPSS score is estimated according to the Passamonti [24]. ° ELN criteria are estimated according to European leukemia network 2017 and 2022 [1,25].

Table S3. Analysis of patient data in AML with developing a karyotype evolution.

Nr.	cytogenetic abnormality	complex karyotype	prior chemotherapy exposure	conditioning intensity	donor type	day-30 response	time to relapse (month)	post-relapse survival (month)
1.	46,XY,del(7)(q32q36)[5]/46,XY[15]	no	7+3, HAM	MAC	MMUD	Mol.gen. CR	6	8
2.	47,XX,+8[19],46,XX[1]	no	2xHAM, TAD9	MAC	MRD	Mol.gen. CR	6	8
3.	46,XY,t(1;3)(p3?6;q26),t(9;22)(q34;q11.2)[3]/ 55,idem,+Y,+8,+10,+12,+15,+19,+20,+21,+der (22)t(9;22)(q34;q11.2)[2]	yes	TKI (Dasatinib)	RIC	MUD	Mol.gen. ° MRD positiv	1	9
4.	45 XX,-7[19]/46,XX[1]	no	CPX-351/Vyxeos	RIC	Haploidentical	Mol.gen. CR	3	6
5.	44,XX,der(4)del(4)(p12)t(4;17)(q21;q12), der(5)t(5;16)(?p12;p12)t(5;16)(q1?4;q21)t(16;9)(q2?4;q3?3),-7,der(9)t(7;9)(?p?;q3?3)+11,-16,-17,-18,der(20)t(4;20)(?q?;q1?3)t(4;7)(?q?;q?31),+21[18]/46,XX[5]	yes	CPX-351/Vyxeos , Decitabine-Venetoclax	RIC	MUD	Mol.gen. CR	13	5
6.	46,XX[25]	no	2x7+3+Midostaurin	MAC	MUD	Mol.gen. CR	22	4
7.	46,XX[10]	no	HAM, Low dose AraC	RIC	MUD	Mol.gen. CR	28	7
8.	45,XY,-5,-14,-16,add(17)(p11.2),-18,del(20)(q11,2) or-	yes	Azacitidine	MAC	MMUD	Mol.gen. CR	3	2

	20,+22,+r(?),+mar1,+mar2 [1]/46,XY,idem,-13, +mar3 [25]/46,XY[1]							
9.	46, XX,inv(16)(p13q22)[15]	no	7+3, HAM	MAC	MUD	Mol.gen. °MRD positiv	10	20
10.	46,XY,del(5)(q14q34),r(7)(p11q11),i(8)(q10), ins(9;14) (p24;q22q32),der(16;21)(p10;q10),-18, +21+r(21)(p11q22)[11]	no	Decitabine	RIC	Hap-loiden-tical	No re-sponse	1	2
11.	45,X,-X,der(3)t(3;16)(p21;p11), der(5)t(5;10)(q14;p12),-7, der(10)t(5;10)(q34;p12),der(12)t(3;12)(q21;q13),del(14)(q21q31),-16, der(20;22)(q10;q10)x3[12]/ 90, XX,-X,-X, der(3)t(3;16)(p21;p11)x2,der(5)t(5;10)(q14;p12)x2,-7,-7, der(10)t(5;10)(q34;p12)x2,-12,-12,der(12)t(3;12)(q21;q13), del(14)(q21q31)x2,-16,-16,der(20;22)(q10;q10)x2, +der(20;22)(q10;q10)x6[3]/46,XX[4]	yes	Decitabine	RIC	MUD	CR	12	1
12.	46,XY,t(8;16)(p11;p13)[15]/46,XY[5]	no	S-HAM	RIC	MRD	Mol.gen. CR	4	1
13.	44,XY,-4,-5,-15,-16,-19,-21,+6mar,3dmins[10]	yes	7+3, HAM	MAC	MUD	relapse	1	1
14.	47,XY,+8 [18] /46,XY[2]	no	Azacitidine, CPX-351/Vyxeos, FLAG-IDA	RIC	MUD	Mol.gen. CR	2	2
15.	46,XY[20]	no	Decitabine-Venetoclax	MAC	MUD	Mol.gen. CR	9	17
16.	46,XX,del(3)(q13),del(5)(q13),-11,add(21)(q22),-22,+r,+mar,2-3dmin[18]	yes	Hy-droxyurea, 7+3+Midosta urin, Azacitidine	RIC	MMUD	relapse	1	3
17.	45,XX,der(3)t(3;13)(q21;q31),t(5;6)(p15;p21),i(5)(p10),-7,der(13)t(3;13)(q21;q31),inv(q21;q26)[12]	yes	AraC-prephase, S-HAM, Clofarabine	RIC	MMUD	CR	2	1
18	46,XY,t(12:17)(q15;23)[20]	no	7+3	MAC	MRD	CR	177	80
19.	46,XX[20]	no	CPX-351/Vyxeos	RIC	MUD	CR	8	2
20.	50,XY,+4,+5,+8,+20[11]/46,XY[6]	yes	7+3	RIC	MUD	CR	3	5

AML, acute myeloid leukemia; 7+3, cytarabine plus daunorubicin; HAM, high-dose cytarabine and mitoxantrone; RIC, reduced-intensity conditioning; TAD9, Thioguanine, Ara-C (Cytarabine), Daunorubicin; TKI, tyrosine kinase inhibitor; CPX-351/Vyxeos (liposomal daunorubicin and cytarabine); S-HAM, sequential high-dose cytarabine and mitoxantrone; FLAG-Ida, fludarabine, Ara-C, G-CSF (Filgrastim), and Idarubicin; MAC myeloablative conditioning; MMUD, mismatched unrelated donor; MRD, matched related donor; MUD, matched unrelated donor; CR, complete remission; CR is confirmed in flow cytometry and NGS; ° MRD positive is confirmed in flow and/or NGS.

Table S4. Analysis of patient data in AML without developing a karyotype evolution.

Nr.	cytogenetic abnormality	complex karyotype	prior chemotherapy exposure	conditioning intensity	donor type	day-30 response	time to relapse (month)	post-relapse survival (month)
1.	46,XX[20]	no	7+3	RIC	MUD	Mol.gen. CR	24	3
2.	46,XX[20]	no	7+3, HAM	RIC	MUD	Mol.gen. ° MRD positiv	5	74
3.	45,XY,-7[20]	no	7+3, MTX i.Th by meningeosis	RIC	MRD	Mol.gen. ° MRD positiv	2	5
4.	46,XX[22]	no	7+3+Midostaurin	MAC	MUD	Mol.gen. ° MRD positiv	1	29
5.	46,XY[20]	no	7+3, mediastinal radiotherapy, HAM	RIC	MUD	Mol.gen. CR	3	3
6.	46,XX[21]	no	Azacitidine, HAM	MAC	MUD	Mol.gen. CR	85	1
7.	47,XY,+8[2]/46,XY[19]	no	Azacitidine	RIC	MUD	Mol.gen. CR	20	22
8.	46,XY[23]	no	Hydroxyurea,7+3	MAC	MRD	CR	12	67
9.	46,XX[20]	no	CPX-351/Vyxeos	RIC	MUD	Mol.gen. CR	4	26
10.	46,XY[20]	no	S-HAM, TAD9, FLAG-Ida, HD-AraC	MAC	MUD	CR	17	45
11.	46,XX[20]	no	TAD9, HAM	RIC	MUD	Mol.gen. ° MRD positiv	4	1
12.	46,XX[13]	no	7+3	RIC	MMUD	Mol.gen. CR	10	4
13.	46,XX[20]	no	7+3,HAM	RIC	MUD	Mol.gen. CR	44	11
14.	48XY,+8,+8,der(12)t(3;12)(q21;p13) [2]/46,XY[20]	yes	prephase Ara-C, CPX-351/Vyxeos	RIC	MUD	Mol.gen. CR	2	27
15.	45,XX,inv(3)(q21q26),-7[20]	no	7+3, low dose Ara-C	RIC	Haploidentical	Mol.gen. ° MRD positiv	5	8
16.	46,XY,inv(16)(p13q22)[20]	no	7+3+Midostaurin, HD-AraC	RIC	MUD	Mol.gen. CR	13	1
17.	46,XY,del(20)(q11q13)[15]/ 46,XY[2]	no	Hydroxyurea, Decitabine-Venetoclax, CPX-351/Vyxeos	MAC	MUD	Mol.gen. CR	2	3

18.	46 XX[22]	no	CPX-351/Vyx-eos , Decita-bine-Veneto-clax	RIC	MUD	Mol.gen. CR	3	3
19.	45 XY,-7[10]	no	7+3, HAM	MAC	MUD	CR	7	2
20.	46,XX [20]	no	Azacitidine-Venetoclax	RIC	MUD	CR	3	10
21.	46,XY [20]	no	Azacitidine	RIC	MUD	CR	51	2
22.	46,XX[6]	no	CPX-351/Vyx-eos	MAC	MUD	CR	72	1

AML, acute myeloid leukemia; 7+3, cytarabine plus daunorubicin; HAM, high-dose cytarabine and mitoxantrone; RIC, reduced-intensity conditioning; TAD9, Thioguanine, Ara-C (Cytarabine), Daunorubicin; Vyxeos (liposomal daunorubicin and cytarabine); S-HAM, sequential high-dose cytarabine and mitoxantrone; FLAG-Ida, fludarabine, Ara-C, G-CSF (Filgrastim), and Idarubicin; MAC, myeloablative conditioning; MMUD, mismatched unrelated donor; MRD, matched related donor; MUD, matched unrelated donor; CR, complete remission; CR is confirmed in flow cytometry and NGS; °MRD positive is confirmed in flow and/or NGS.
